# Investigation of Surface Morphology of 6H-SiC Irradiated with He^+^ and H_2_^+^ Ions

**DOI:** 10.3390/ma11020282

**Published:** 2018-02-11

**Authors:** Qiang Shen, Guang Ran, Wei Zhou, Chao Ye, Qijie Feng, Ning Li

**Affiliations:** 1 College of Energy, Xiamen University, Xiamen 361102, China; shenqiang1989@126.com (Q.S.); kim.yc@foxmail.com (C.Y.); 2 China Academy of Engineering Physics, Mianyang 621900, China; zhouwei_801202@163.com (W.Z.); fengqijie@caep.cn (Q.F.)

**Keywords:** SiC, surface morphology, irradiation, blister

## Abstract

Light ion implantation is one of the important procedures of smart cut for SiC-based semiconductor fabrication. This work investigated the surface morphologies and microstructures of single crystal 6H-SiC irradiated by one or both of H_2_^+^ and He^+^ ions at room temperature and then annealed at specific temperatures. Blisters evolved from the coalescence of H nanocracks were formed in the H_2_^+^ and He^+^+H_2_^+^ irradiated sample surface, while circular ripples originated from the pressure release of helium bubbles after high temperature annealing were formed in the He^+^ irradiated sample surface. The lateral radius *a* of the blisters in the irradiated sample with low H_2_^+^ fluence was larger than that in the irradiated sample with high H_2_^+^ fluence and with He^+^+H_2_^+^ ions. About 8–58% of implanted H atoms contributed to the formation of the blisters. Compared with other irradiated samples, the ratio of *w*_0_/*a* and the density of the blisters in the He^+^+H_2_^+^ irradiated samples were largest. The stress field of the blisters was simulated using finite element method and the inner pressure in the blisters was also calculated. The corresponding mechanism was analyzed and discussed.

## 1. Introduction

SiC-on-insulator (SiCOI) structures are considered as the most promising semiconductor materials for high-speed, high-power and high-temperature microelectronic applications due to their superior properties, i.e., high temperature physical and chemical stability, low power dissipation, and high radiation resistance [1,2]. Smart cut is an efficient and economical technology for manufacturing SiCOI structures. The process contains three main steps: (1) light ion implantation; (2) wafer bonding; and (3) fracture to achieve thin layer transfer [3,4,5]. H and He ions are usually used as light ion implantation. However, surface features induced by ion implantation, for sample, blistering and exfoliation, have a great influence on the layer transfer and the properties of SiCOI structures [6]. Therefore, it is of great importance to investigate the irradiation-induced defects and their recovery process during H and He ion implantation and then annealing. 

Surface blistering has been widely investigated in the various kinds of materials, e.g., Si [7,8], W [9,10], Ge, GaAs [11], etc. However, few works study the blisters in SiC materials after gas ion irradiation. The literature on SiC irradiated with H, D and He ions reported the formed surface blisters [12,13]. The relationship between the efficiency of H blistering in the SiC and H ion irradiation parameters, i.e., ion fluence and irradiation temperature, were also studied [14,15]. The average size of H blisters decreased with the increase H^+^ ion fluences from 6 × 10^16^ H^+^/cm^2^ to 1 × 10^17^ H^+^/cm^2^ while the number density of blisters increased distinctly when the sample was irradiated with 180 keV H^+^ ions and annealing at 950 °C for 30 min [14]. Li [15] found that the average diameters of H blisters increase with the increase of irradiation temperature in the range from room temperature (RT) to 773 K. The evolution of amorphous structure in the irradiated layer also affected the formation of blisters during the annealing process [16]. Other influential blistering parameters, e.g., ion species and co-effects of several kinds of ions, should be further researched. Igarashi [13] compared the shape difference between H-blisters and He-blisters and reported the lateral radius of H-blisters was larger than that of He-blisters, and the opposite for the vertical deformation of He-blisters. However, the essential reason for the difference needs to be further investigated. In most inert gas ion irradiated materials, blistering is believed to be evolved from the accumulation of gas atoms and coalescence of gas bubbles [17,18,19]. However, in the H irradiated SiC, H atoms were able to terminate the broken Si-C bonds and then diffused in the form of H_2_, CH_4_ or SiH_4_, where the lateral propagation of H cracks and blister deformation were inevitably affected [13,19,20]. Thus, the mechanism of blistering in SiC is not completely understood. Meanwhile, the exfoliation of blisters was observed after blistering while few works were done to explore the mechanism.

In the present work, single crystal SiC irradiated by one or both of H_2_^+^ and He^+^ ions and then annealing were done to investigate the surface features and microstructures. To get a better understanding of the mechanism of blistering and exfoliation, the stress field of the blisters was simulated using finite element method and the inner pressure in the blisters was also calculated. The corresponding mechanism was analyzed and discussed. 

## 2. Experiments

**Figure 1:**
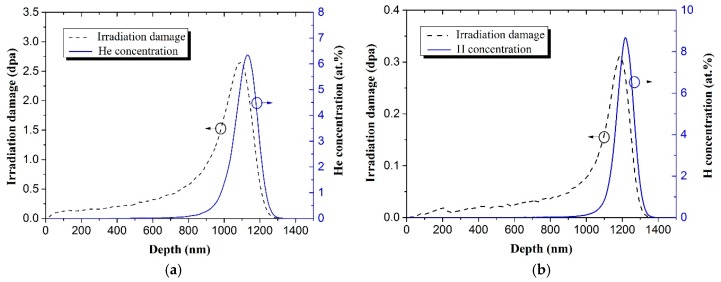
The profiles of irradiation damage and ion concentration of: 400 keV He^+^ with a fluence of 1 × 10^17^ He^+^/cm^2^ (**a**); and 200 keV H_2_^+^ with a fluence of 5 × 10^16^ H_2_^+^/cm^2^ (**b**), simulated by SRIM 2013 software with quick mode.

The single crystal 6H-SiC samples with [0001] crystal direction from MTI Corporation were irradiated by H2+ or/and He+ ions at room temperature and subsequently annealed at some given experiment conditions. The experiment conditions of ion irradiation and annealing were listed in Table 1. The displacement per atom (dpa) and implanted ion concentration of H_2_^+^ and He^+^ were simulated by SRIM 2013 software (SRIM 2013, http://www.srim.org/) with quick mode in order to achieve similar peak depth of H_2_^+^ and He^+^ ion concentration, as shown in Figure 1. The displacement energies of C and Si were assumed to be 20 eV and 35 eV, respectively. The concentration peaks of H_2_^+^ (200 keV) and He^+^ (400 keV) ions appeared at the depth of ~1.1 μm and ~1.2 μm, respectively. The irradiated and annealed sample surface was characterized by ZeGage NewView™ 6300 3D (three dimension) optical profiler (3D OP) (Zygo Corp., Middlefield, CT, USA) with 0.1 nm vertical accuracy. Cross-sectional structures of the blisters in the sample surface were examined by cross-sectional scanning electron microscopy (X-SEM) and cross-sectional transmission electron microscopy (X-TEM). The sample surface used to X-SEM analysis was protected by epoxy glue and then polished by the diamond paper carefully to avoid the damage of blisters. The preparation methods of X-TEM samples could be found in our previous work [21].

## 3. Results and Discussion

**Figure 2:**
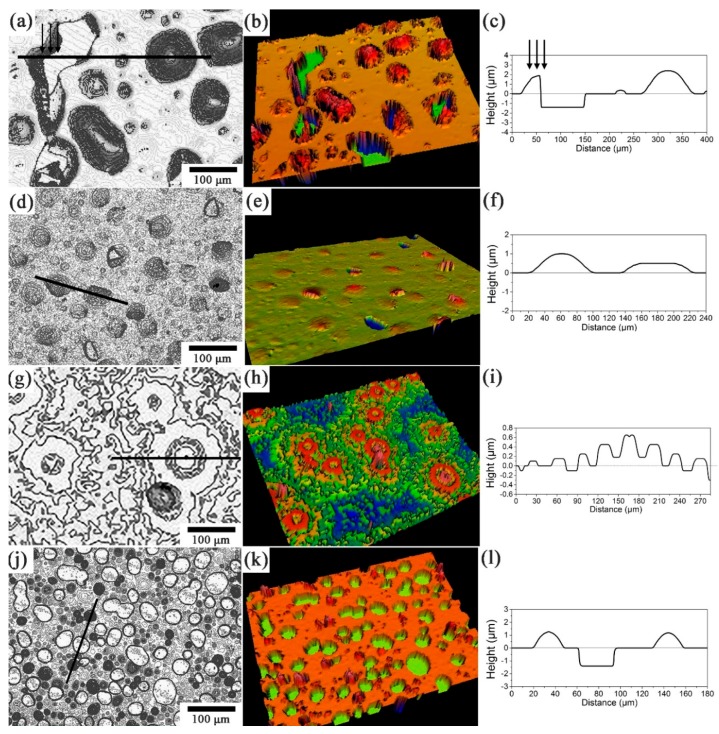
2D morphologies, 3D topographies and the cross-sectional profiles of the blisters in the irradiated and annealed 6H-SiC sample surface: (**a**–**c**) Sample 1; (**d**–**f**) Sample 2; (**g**–**i**) Sample 3; and (**j**–**l**) Sample 4.

Figure 2 shows the morphologies and topographies of the irradiated and annealed SiC surface characterized by 3D optical profiler. Figure 2a,b presents the two-dimensional (2D) morphologies and 3D topographies of 6H-SiC irradiated with a fluence of 5 × 10^16^ H_2_^+^/cm^2^ and then annealed at 900 °C for 30 min. Blisters are formed on the sample surface. Figure 2c is the cross-sectional profile of the blisters in the 2D OP image along the straight line in Figure 2a. The corresponding morphology, topography and the cross-sectional profile of the blisters in Sample 2 are shown in Figure 2d–f, respectively. Compared with the test results of Sample 1, the size of the blisters is decreased with increasing H_2_^+^ ion fluence. The density of the formed blisters is obviously increased when the He^+^ irradiated sample was then implanted with H_2_^+^ ions (Sample 4) (compare Figure 2a,b with Figure 2j,k). Meanwhile, the blisters will exfoliate from SiC matrix when they grow up to a critical size, which could be attributed to the high stress concentration. Approximately 50% blisters exfoliate from the surface of the He^+^ and H_2_^+^ irradiated sample while very few blisters exfoliate from the H_2_^+^ irradiated sample after annealing for 30 min at 900 °C. Furthermore, the blisters in the He^+^ and H_2_^+^ irradiated sample exfoliate along the boundary between the blisters and SiC substrate, as shown in Figure 2h. However, the exfoliation shape of blisters in the H_2_^+^ irradiated samples is rather irregular. Figure 2c shows a partially exfoliated blister. The cross-sectional profile of the blisters indicates that a steep fracture cliff has been formed after annealing, as indicated by arrows in Figure 2a,c, which is due to part of the blister remaining, while the other part is exfoliated. The depth of the crater is uniform and corresponding value is approximately 1.4 μm.

The surface morphologies and topographies of the 6H-SiC irradiated by He^+^ ions with a fluence of 1 × 10^17^ He^+^/cm^2^ are obviously different from other three experiment conditions. Surface features start to be formed when the annealing temperature is over 1200 °C. After be irradiated with a fluence of 1 × 10^17^ He^+^/cm^2^ and then annealed at 1500 °C for 30 min, some circular ripples are formed on the sample surface, as shown in Figure 2j–l.

**Figure 3:**
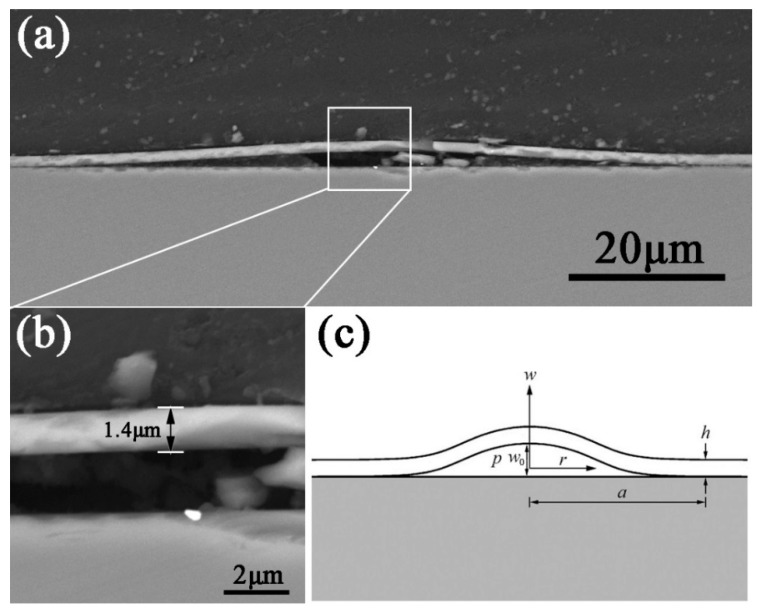
(**a**) X-SEM image of a typical blister in Sample 1; (**b**) the thickness of the blister; and (**c**) the simplified model of the blister.

X-SEM images of a typical blister in Sample 1 show the cross-sectional morphology (Figure 3a). The blister shape is considered pure bent and the curvature is three-dimensionally successive. The thickness of the blister is about 1.4 μm, as shown in Figure 3b. The shape of the blisters in the H_2_^+^ and He^+^ irradiated samples can be considered as circular-plate shape. The function used to describe the blister profile (Normal to the sample surface) can be expressed as [22]:

(1) $$w_{r}=w_{0}{\left(1-\frac{r^{2}}{a^{2}}\right)}^{2}$$

where *a*, *r*, *w*_0_ and *w_r_* are the maximum radius of the bottom circle of the blisters, radius, the maximum amplitude value at the center of the blister and the amplitude of the blister at a given radius *r*, respectively, which are indicated in the simplified model of the blister, as shown in Figure 3c.

**Figure 4:**
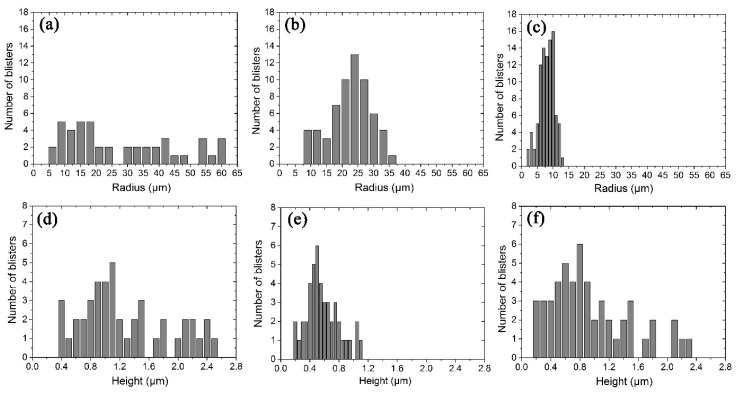
The distribution characteristics of the radius and height of the blisters in the cross-sectional: (**a**,**d**) Sample 1; (**b**,**e**) Sample 2; and (**c**,**f**) Sample 4.

According to the simplified model of the blister in Figure 3c, the values of lateral radius (*a*), the vertical deformation height (*w*_0_) and the thickness of the blisters were measured in irradiated Samples 1, 2, and 4. The statistical results of the distribution of *a* and *w*_0_ are shown in Figure 4. The radius of the blisters in Sample 1 varies from 5 to 65 μm and the numbers of the blisters have a homogeneous distribution in this range. The vertical deformation height varies in the range of 0.3–2.5 μm. As the H_2_^+^ ion fluence is increased, the radius become smaller in Sample 2. The vertical deformation height decreases significantly in Sample 2. Irradiated with He^+^ and H_2_^+^ ions in Sample 4, the lateral radius of the blisters is smallest and distributed in a narrow range of 1–15 μm. However, the vertical deformation height of the blisters in Sample 4 does not change too much compared to that of Sample 1. That means the ratio (*w*_0_/*a*) of the blisters in Sample 4 is largest. The number distributions of *a* and *w*_0_ in Figure 4b–d are very close to normal distribution while the number in Figure 4a has a homogeneous one. For the further calculation of stress and inner pressure, average values of the parameters *a*, *w*_0_ and *h* are calculated and shown in Table 2. The average values are calculated with Gaussian fitting for the data in Figure 4b–d and averaging method in Figure 4a. Observed from the morphologies and topographies of sample surface, the exfoliated blisters are relatively few in the H_2_^+^ irradiated samples. However, nearly half of blisters exfoliate from the He^+^ and H_2_^+^ irradiated sample surface when they grow to a critical size. The average radius (*a*) of the exfoliated blisters in Sample 4 is approximately 15.6 μm while the vertical deformation height (*w*_0_) is unavailable because only the concave pits stayed on the sample surface.

The inner gas pressure, stress distribution and implanted ion fluence in the blisters were estimated by finite element method (FEM) simulation with ABAQUS software (ABAQUS 6.13, Dassault Systèmes Corp., Providence, RI, USA) [23,24,25]. An axisymmetric system is used. In fact, ion irradiation inevitably changes the microstructure and then affects the mechanical properties of SiC [26,27]. During FEM simulation, SiC is considered as ideal brittle material and only elastic deformation is taken into account. Poisson’s Ratio (*v*) and yield strength of SiC sample are set as 0.14 [28] and 21 GPa [29], respectively. According to our TEM observation results of the as-irradiated samples, the irradiated layer and sandwich structure are formed near the sample surface. To simplify the irradiation effect, the sample is divided into three layers during FEM analysis: the surface layer, the irradiated layer and the substrate layer. The thickness measured from cross-sectional TEM images and elastic modulus referenced from the literatures of these three layers in Samples 1, 2, and 4 are listed in Table 3. Then, an ultra-thin crack is introduced at the depth corresponding to the measured depth of blisters (~1.4 μm). Finally, the gas pressure value will be obtained when the deformation height of surface layer meets the experimental value at the center of the blister (*w*_0_) of the blisters [23,24]. Additionally, the inner gas pressure is also calculated according to the theoretical elastic model developed by Timoshenko [22]. The elastic modulus *E* in Equation (2) is modified with thickness-weighted calculation according to the divided layer structure. The calculating equation is shown as follows:

(2) $$E_{m}=\frac{E_{s}\cdot T_{s}+E_{i}\cdot T_{i}}{T_{s}+T_{i}}$$

where, *E_m_*, *E_s_*, *T_s_*, *E_i_*, and *T_i_* represent the modified value of elastic modulus used for calculating the inner pressure of blisters theoretically, elastic modulus of surface layer, thickness of surface layer, elastic modulus of irradiated layer and thickness of irradiated layer, respectively. The values of *E_s_*, *T_s_*, *E_i_*, and *T_i_* are found in Table 3. The values of *E_m_* were calculated to be 489.4, 481.8 and 435.9 GPa for Samples 1, 2, and 4, respectively. The inner gas pressure (*p*) can be calculated according to following equation [30]:

(3) $$p=\frac{16E_{m}h^{3}w_{0}}{3a^{4}\left(1-v^{2}\right)}$$

where, *p*, *E_m_*, *h*, *w*_0_, *a*, and *v* represent the pressure, the modified value of elastic modulus, the thickness of the blisters, the vertical deformation of blisters, the radius of bottom circle of blisters and Poisson’s ratio, respectively.

Table 4 lists the gas pressure inside the blisters calculated from theoretical model and FEM simulation. The data show that the FEM simulation results are comparable with these obtained from theoretical model. For the only H_2_^+^ irradiated and then annealed Samples 1 and 2, the inner gas pressure stays at tens of MPa. However, in the He^+^ and H_2_^+^ irradiated and annealed Sample 4, the inner pressure researches several hundreds of MPa. Muto’s work indicated that the inner gas pressure was about 400 MPa in the Si sample after irradiation with a fluence of 1 × 10^18^ H^+^/cm^2^ [23]. Hong gave 10–1000 MPa pressure range in the Si sample irradiated with a fluence of 1 × 10^17^ H^+^/cm^2^ [33]. The calculated pressures in our work is reasonably in this range. 

**Figure 5:**
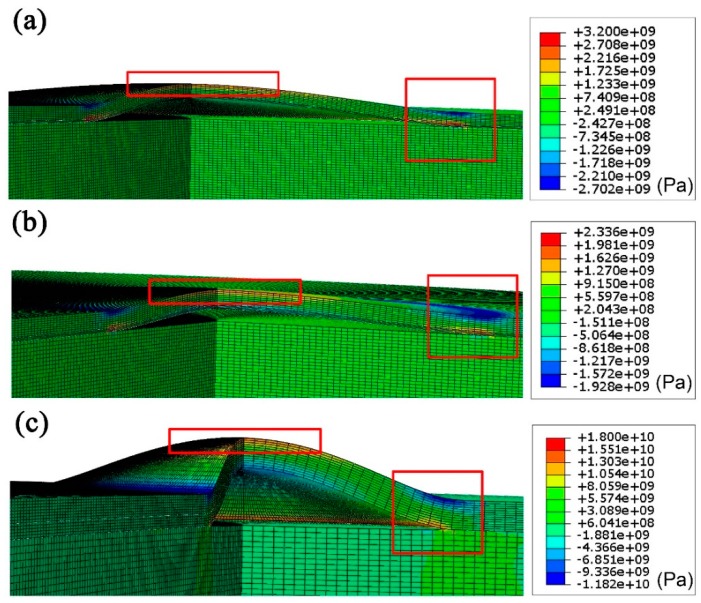
FEM simulation results showing the stress distribution in the blisters in irradiated: (**a**) Sample 1; (**b**) Sample 2; and (**c**) Sample 4.

Figure 5 shows the stress contours of the blisters on the surfaces of Samples 1, 2, and 4 derived from FEM. The stress concentrates at the center and the boundary of blisters, marked with red boxes in Figure 5. The maximum values of concentrated stress are distributed on the lower face of blisters and the extreme stress (*r* = 0 and *r* = *a*) on the lower surface of the blister, as listed in Table 4. The concentrated stress stays small for the H_2_^+^ irradiated samples. In the He^+^ and H_2_^+^ irradiated blisters, the stress at the boundary increased rapidly to 15.2 GPa. The FEM simulation also reveals that when the inner pressure for the exfoliated blisters (with lateral radius of ~15.6 μm) reaches 372 MPa, the stress would exceed the theoretical yield strength (equals to fracture strength of brittle materials) of 21 GPa [29]. Consequently, the formed blisters can easily fracture and exfoliate along the boundary between the blisters and SiC matrix in Sample 4, which is the reason that approximately ~50% blisters exfoliate from the H_2_^+^ and He^+^ irradiated sample surface. 

The number of gas molecules inside the blister are calculated using van der Waals equation as following:

(4) $$n^{3}-\frac{V}{\gamma }n^{2}+\frac{\gamma pV^{2}+RTV^{2}}{\beta \gamma }n-\frac{PV^{3}}{\beta \gamma }=0$$

where, *n*, *V*, *P*, *T* and *R* are the number of molecules, blister volume, pressure, kelvin temperature, and gas constant, respectively. The parameters “*β*” and “*γ*” in Equation (3) are van der Waals constants (for H_2_: *β* = 0.245 × 10^−6^ atm·m^6^/mol^2^ and γ = 0.0267 × 10^−6^ atm·m^6^/mol^2^; and for He: β = 0.034 × 10^−6^ atm·m^6^/mol^2^ and γ = 0.0238 × 10^−6^ atm·m^6^/mol^2^). Considered the delamination depth of blisters, the parameters of H_2_ are used in Equation (3). The blister volume can be obtained from Equation (4):

(5) $$V=\frac{\pi a^{2}w_{0}}{3}$$

Table 5 lists the calculated molecules (*n*) and ion fluences (*N*) inside the blisters. The pressure used in Equation (3) is calculated by FEM simulation, as shown in Table 3. In the H_2_^+^ irradiated Sample 1, the percentage of H atoms inside the blisters is as high as 58% of total implanted H atoms. In the H_2_^+^ of 1 × 10^17^ H_2_^+^/cm^2^ irradiated samples, the calculated hydrogen molecule and fluence are smaller. Only 8% of implanted H_2_^+^ ions contribute to the blister formation. Previous literature reported that 20–40% of total implanted gas ions were contributed to the H-blisters in the Si [34] and W [35,36] materials. Thus, the calculated molecules in the blisters are reasonable in the present work.

**Figure 6:**
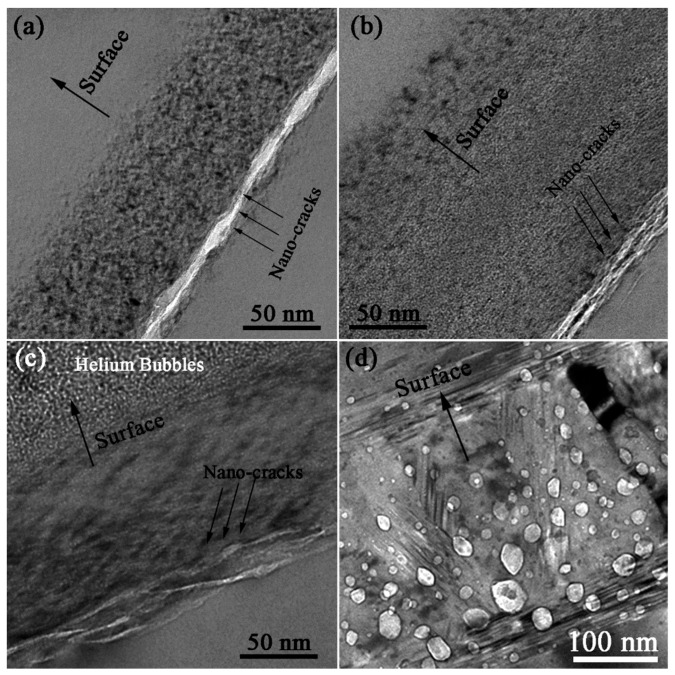
Bright field X-TEM images of: (**a**) Sample 1; (**b**) Sample 2; (**c**) Sample 4; and (**d**) Sample 3.

Figure 6a–c shows the bright field X-TEM images of SiC Samples 1, 2, and 4, respectively. It can be observed that a thin layer distributed with gas bubbles and nanocracks (defined as irradiated layer) with approximately 70 nm thickness were formed in SiC sample. The nanocracks mainly distribute in the approximately 1.4 μm depth. However, with increasing H_2_^+^ ion fluence, the width of irradiated layer is increased. In Sample 2, the width of irradiated layer is up to about 170 nm, which is about two and a half fold of that in Sample 1. In addition, the middle region of bubble layer maintains amorphous state after annealing for 30 min. The distribution width of the nanocracks in the He^+^ and H_2_^+^ irradiated sample is approximately 20 nm, which is larger than that in the H_2_^+^ irradiated samples. A large amount of helium bubbles are formed far away the nanocracks region at a depth of ~1.2 μm as shown in Figure 6c. 

The distribution depth of these nanocracks is exactly equal to the thickness of the blisters, which indicates that the blisters originate from these nanocracks. The blister evolution should include the growth of the maximum radius of the blister (*a*) and the maximum amplitude deformation value at the center of the blister (*w*_0_). The increment of *a* value can be achieved through the interconnection and coalesce of these nanocracks. The density and the slender shape of H nanocracks affect the coalescence of these H nanocracks. In the H_2_^+^ irradiated samples, the concentrated distribution characteristics of the formed nanocracks make the blister grow easily, as shown in Figure 6a. When the samples were irradiated with higher H_2_^+^ fluences, the density of nanocracks is larger. These blisters are more likely to be connected and the inner pressures between the blisters were balanced before the blister grew bigger. Thus, the average size of the blisters of Sample 2 (including *a* and *w*_0_) is relatively smaller than that of Sample 1. Additionally, the existence of amorphous region and the inadequate annealing of the damaged layer may also affect the evolution of the blisters [16]. The He^+^ and H_2_^+^ irradiated Sample 4 has larger number density of the blisters. Meanwhile, the blisters have extremely high inner pressure as listed in Table 4. The wider vertical distribution of H nanocracks makes the lateral propagation and coalesce of H nanocracks difficult. These explain that the lateral radius *a* (9.6 μm) of Sample 4 is smaller than that of Samples 1 and 2. Meanwhile, the He^+^ irradiation reduces the elastic modulus of the irradiated layer, which gives a relatively big value of vertical deformation *w*_0_. Therefore, larger ratio of *w*_0_/*a* of the blisters is achieved. 

Figure 6d shows the microstructure of bright field X-TEM image of Sample 3 (only He^+^ ion irradiation). Large helium bubbles, which have radii of several tens of nanometers, are formed in the He concentration peak region at the depth of ~1.2 μm. The growth of helium bubbles driven by the mobility of vacancies become significant when the annealing temperature is over 1000 °C [37,38]. The formation of circular ripples in the sample surface, as shown in Figure 3g–i, is believed to initiate from the coalescence of these bubbles. Our previous research also revealed a rapid growth of the helium bubbles during the first 30 min as the annealing temperature was above 1200 °C [21]. It can be speculated that the release of extremely high internal pressure in the bubbles is achieved in this annealing stage by creating an exit in the center area of bubbles. This phenomenon is similar to volcanic eruption, which induces the formation of the circular ripple structure that is proved by the surface topography of 3D OP analysis. To the best of our knowledge, the circular structure of surface ripples is first observed in the He^+^ irradiated and then annealed SiC material. However, further research on the detailed mechanisms and building models to explain this formation on the surface topography is needed.

## 4. Conclusions 

The microstructure of the single crystal 6H-SiC samples with [0001] crystal direction irradiated by one or both of H_2_^+^ and He^+^ ions and then annealed were investigated by 3D optical profiler, scanning electron microscopy and transmission electron microscopy. Blisters evolved from the coalescence of H nanocracks were formed in the H_2_^+^ and He^+^+H_2_^+^ irradiated sample surface, while the circular ripples originated from the pressure release of helium bubbles after high temperature annealing were formed in the He^+^ irradiated sample surface. The lateral radius *a* of the blisters in the irradiated sample with low H_2_^+^ fluence was larger than that in the irradiated sample with high H_2_^+^ fluence and with He^+^-H_2_^+^ ions. Fracture and exfoliation of the blisters in the He^+^+H_2_^+^ irradiated sample were attributed to the stress concentration that exceeded the theoretical yield strength. Compared with other irradiated samples, the ratio of *w*_0_/*a* and the density of the blisters in the He^+^-H_2_^+^ irradiated samples were largest, which should be attributed to: (i) the widened distribution of H nanocracks in vertical direction; and (ii) the He^+^ implantation induced the reduction of elastic modulus of the irradiated layer. In addition, about 8–58% of implanted H atoms contributed to the formation of the blisters. 

## Figures and Tables

**Table 1:** Irradiation conditions of H_2_^+^ and He^+^ ions.

Sample	200 keV H_2_^+^		400 keV He^+^	Annealing Conditions
	5 × 10^16^ H_2_^+^/cm^2^	1 × 10^17^ H_2_^+^/cm^2^	1 × 10^17^ He^+^/cm^2^	
1	√	−	−	at 900 °C for 30 min
2	−	√	−	at 900 °C for 30 min
3	−	−	√	at 1500 °C for 30 min
4	√	−	√	at 900 °C for 30 min

**Table 2:** The parameter values of the blisters in the irradiated and annealed 6H-SiC.

Sample	*a* (μm)	*w*_0_ (μm)	*h* (μm)
1	28.7 ± 13.0	1.1 ± 0.4	1.4 ± 0.1
2	21.6 ± 8.0	0.5 ± 0.2	1.4 ± 0.1
4	9.6 ± 4.0	0.9 ± 0.4	1.4 ± 0.1

**Table 3:** The structure parameters used in FEM analysis.

Sample	Thickness (μm)			Elastic Modulus (GPa) [31,32]
	1	2	4	
Surface layer	1.200	1.15	0.850	520
Irradiated layer	0.20	0.250	0.550	306
Substrate layer *	30	30	30	520

* The same thickness ~30 μm of substrate layer was used for all the samples during FEM modeling.

**Table 4:** Inner gas pressure and stress from theoretical calculation and FEM simulation.

Sample	Gas Pressure, *p* (MPa)		${\left(\sigma_{r}\right)}_{r=0}$ (GPa)	${\left(\sigma_{r}\right)}_{r=a}$ (GPa)
	Theoretical Calculation	FEM Simulation		
1	11.8	13.4	0.74	2.8
2	16.5	14.2	0.8	2.1
4	689.5	573	6.3	15.2

**Table 5:** Average values of blisters estimated by FEM.

Sample	*V* (m^3^)	*S* (m^2^)	*n*	*N* (Ions/cm^2^)
1	9.5 × 10^−16^	2.6 × 10^−9^	7.6 × 10^11^	2.9 × 10^16^
2	2.4 × 10^−16^	2.9 × 10^−10^	2.1 × 10^11^	8.0 × 10^15^
4	8.7 × 10^−17^	1.5 × 10^−9^	1.2 × 10^12^	4.7 × 10^16^
